# The little molecules that could: a story about microRNAs in neural stem cells and neurogenesis

**DOI:** 10.3389/fnins.2012.00176

**Published:** 2012-12-07

**Authors:** Grace E. Asuelime, Yanhong Shi

**Affiliations:** ^1^Department of Neurosciences, Beckman Research Institute of City of HopeDuarte, CA, USA; ^2^Department of Biological Sciences, California State Polytechnic University PomonaPomona, CA, USA

There are many biological processes occurring in nature with dynamic complexity: neurogenesis is one such process. The special topic of “microRNAs in Neural Stem Cells (NSCs) and Neurogenesis” in Frontiers in Neuroscience discussed diverse roles microRNAs (miRNA) play in neurogenesis through a series of review articles and original research articles. This editorial serves to highlight these appealing articles, which build a comprehensive story about the many different roles miRNA molecules play in NSCs and neurogenesis.

Neurogenesis involves the generation of newborn neuronal cells from NSCs and progenitor cells in the mammalian brain. The main steps of neurogenesis include NSC self-renewal, neural progenitor cell proliferation, neuronal commitment, migration, maturation, and integration (Shi et al., 2010). Neurogenesis is highly active during development when new neurons are formed and integrated into the growing brain. Neurogenesis continues to occur in the brain throughout adulthood within two discrete adult neurogenic niches, the hippocampal dentate gyrus and the subventricular zone (Lois and Alvarez-Buylla, 1994; Eriksson et al., 1998).

In recent years, much attention has been focused on the impact miRNAs have on the process of neurogenesis. miRNAs are single-stranded, non-coding molecules that typically range between 21 and 24 nucleotides in length. They are post-transcriptional regulators that are endogenously expressed and bind to complementary sequences of messenger RNA targets. miRNAs regulate multiple processes including: development, cell proliferation and differentiation, growth and neurogenesis (Ambros, 2004; Bartel, 2004). Though small in length, miRNAs control gene expression through targeting many downstream targets. In a recent review, miRNAs are introduced as an important player in the regulation of embryonic stem cells and neurogenesis (Kawahara et al., 2012). Emphasis is placed on components of miRNA biogenesis, such as Dicer, Drosha, DGCR8, Lin-28, and other related proteins involved in neurogenesis; some of which have been linked to cancer, fragile × syndrome, and nervous system disorders (Kawahara et al., 2012). The review written by Lang and Shi highlighted the dynamic roles that miRNAs play in multiple steps of neurogenesis including: NSC proliferation, self-renewal, neuronal differentiation, maturation, and dendritic spine morphogenesis (Lang and Shi, 2012). This review covered key miRNA regulators in neural development and adult neurogenesis. It summarized the role of miR-9, miR-124, miR-137, miR-184, and let-7 in NSC proliferation and differentiation, miR-125b and miR-128 in neuronal differentiation and maturation, and miR-132, miR-134, miR-138 in dendritic spine morphogenesis (Lang and Shi, 2012).

Switching gears from neuronal differentiation, Barca-Mayo and Lu discussed miRNAs important for glial-lineage fate specification with an emphasis on the fine-tuning of oligodendrocyte development (Lu and Barca, 2012). The authors elaborated on the roles of miRNAs, particularly miR-219, miR-338, and miR-138, in regulating oligodendrocyte differentiation and maturation. Also highlighted in this article is a discussion concerning the potential use of miRNAs as disease biomarkers for nervous system diseases, such as multiple sclerosis, owing to a greater stability of miRNAs in comparison to messenger RNAs (Lu and Barca, 2012).

As discussed by Schouten et al., miRNAs have been implicated in aging-associated cognitive decline, synapse formation, and the effects of circulating levels of steroid hormones (Schouten et al., 2012). Within this framework, the ability of newborn neurons to functionally integrate into hippocampal circuits could be partly attributed to the activity of miR-132. Furthermore, the authors gave a compelling account of miRNAs in adult hippocampal neurogenesis, along with their effects on target genes and the potential influence these interactions have on neural development disorders, such as Rett syndrome and autism.

A discussion concerning neurogenesis would not be complete without taking a look at the epigenetic mechanisms regulating gene expression important for NSC maintenance and fate specification. In their review, Jobe et al. explored the ability of non-coding RNAs to “crosstalk” with other epigenetic mechanisms—namely DNA methylation and histone modification. The authors paid particular attention to the contributions of epigenetic mechanisms to NSC regulatory networks in adult neurogenesis (Jobe et al., 2012). Furthermore, this review raises a discussion concerning how neuronal activities, inflammation, stress, and diseases lead to changes in epigenetic states.

While there are many tools offering a “straight-forward” approach to miRNA transcriptional profiling, such as miRNA arrays, PCR, and Northern blotting, functional studies on miRNA remain technically challenging (Akerblom et al., 2012). For instance, Dicer knockout studies suggest critical roles for miRNA in neurogenesis, but the results are difficult to interpret because knockout of Dicer affects all miRNAs, not just the miRNAs of interest. In addition, the stability of mature miRNAs makes conditional Dicer knockout studies difficult to control temporally. This being said, in their review, Akerblom et al. discussed examples taken from studies done on critical regulatory miRNAs in neurogenesis, including miR-124, miR-9, and Let-7 family members (Akerblom et al., 2012).

In an interesting original research article, Chen and Wichterle performed conditional knockout of Dicer in motor neuron progenitors in order to elucidate a role for miRNAs in the regulation of post-mitotic neurons and fate specification of different motor neuron subtypes in each segment of the developing spinal cord (Chen and Wichterle, 2012). Their studies revealed that the requirement for Dicer function in motor neurons is subtype-specific and that miRNAs are important for proper motor pool specification and the maintenance of motor neurons (Chen and Wichterle, 2012).

In another original research article included in this series, a miRNA sponge was used to block the silencing activity of miR-9 to study the functional role of miR-9 (Otaegi et al., 2012). The authors demonstrated that blocking endogenous miR-9 allows for stronger expression of FoxP1 and a mild reduction in Lhx3-expressing motor neurons, suggesting the involvement of miRNAs in the fine-tuning process of motor neuron subtype identity specification (Otaegi et al., 2012).

Using miRNA genome-wide array profiling combined with bioinformatics analysis, Gao et al. identified a collection of miRNAs dynamically regulated by the zinc-finger protein, REST, during neuronal differentiation of primary mouse NSCs. In this study, the authors found that REST is critical for the proper expression of certain miRNAs under varying differentiation conditions, which in turn, provides insight into the REST-regulated stage-specific expression of miRNAs during NSC-neuronal differentiation (Gao et al., 2012). Studies such as these help us to unfold the role that miRNAs play in neurogenesis.

In conclusion, the study of neurogenesis is a poignant area of study, particularly because of the difficulties associated with modeling human brain diseases and injuries. Studies of key molecules that regulate the dynamic interactions in the brain, particularly miRNAs, present an opportunity to control and fine-tune NSC populations and cell fate conversion, as they are capable of intricate balance and regulation. Together, these articles reflect a sophisticated and exciting story of the dynamic roles miRNAs play in NSCs and neurogenesis. Further elucidation of the roles of small non-coding RNAs in brain development will serve as a powerful tool in brain disease modeling and therapeutics.
